# Interaction of human erythrocyte catalase with air*–*water interface in cryoEM

**DOI:** 10.1093/jmicro/dfab037

**Published:** 2022-02-18

**Authors:** Shaoxia Chen, Jade Li, Kutti R Vinothkumar, Richard Henderson

**Affiliations:** MRC Laboratory of Molecular Biology, Francis Crick Avenue, Cambridge CB2 0QH, UK; MRC Laboratory of Molecular Biology, Francis Crick Avenue, Cambridge CB2 0QH, UK; MRC Laboratory of Molecular Biology, Francis Crick Avenue, Cambridge CB2 0QH, UK; National Centre for Biological Sciences (NCBS), Tata Institute of Fundamental Research, Bellary Road, Bengaluru 560065, India; MRC Laboratory of Molecular Biology, Francis Crick Avenue, Cambridge CB2 0QH, UK

**Keywords:** air–water interface, human erythrocyte catalase, cryoEM, CHAPSO detergent, 3D structure

## Abstract

One of the key goals in single-particle cryo-microscopy is to obtain a uniform distribution of particle orientations, so that the three-dimensional structure has isotropic resolution in Fourier space. A common problem arises from the interaction of protein molecules with the air–water interface that exists on both surfaces of the thin film of liquid that is formed prior to plunge-freezing into liquid ethane. Some proteins and other macromolecular complexes are disrupted by interaction with the air–water interface. Other proteins or macromolecules either become concentrated through their interaction with the interface or are excluded because they bind strongly to some other part of the grid or the filter paper used in blotting. In this paper, the interaction of human erythrocyte catalase with the air–water interface is investigated and minimized by the addition of certain detergents. Detergents can form an amphipathic monolayer at the air–water interface that creates a barrier and leaves the molecules free to adopt a variety of orientations, thus facilitating the 3D structure determination. These results suggest that further characterization and development of detergents for cryo-microscopy plunge-freezing would be useful.

## Introduction

Since the introduction of plunge-freezing to produce thin films of amorphous ice by Dubochet and his colleagues [1–3], its use for three-dimensional structure determination of a wide range of biological structures by electron cryo-microscopy (cryoEM) has expanded greatly. The number of structures being deposited in the Protein Data Bank each year using cryoEM is still growing exponentially [4]. In contrast to the outstanding success of the method, frequently called ‘the resolution revolution’ [5], there is a less appreciated barrier, which is the difficulty of preparing a thin film with an even distribution of intact structures of interest that display enough different orientations to allow adequate sampling of the Fourier components in reciprocal space [6–10]. Many strategies have been developed to tackle this requirement, but no single approach has yet been developed that is applicable to every specimen. In some cases, often involving particles with high symmetry such as icosahedral viruses, it is straightforward, but in other cases there is such severe preferential orientation due to interaction with the surface of the thin film that measures such as recording images of highly tilted grids is the only solution in obtaining a wider range of views [6,7]. A typical approach is simply to keep trying different conditions so that eventually a single grid is obtained with a sufficiently good distribution to be able to collect enough images for structure determination, and the problem is not investigated further. Nevertheless, it would still be very desirable to be able to develop a general procedure that would work for any molecule of interest.

The number of particles to expect in cryoEM images of samples prepared by plunge-freezing based on the concentration of the protein in the drop prior to blotting is quite straightforward to calculate and has been emphasized previously [11–13]. Frequently however, when grids are examined, there are either many fewer or many more particles than expected. This is due either to adsorption of the molecules by the filter paper or the carbon or gold supporting films which decreases the concentration of free molecules, or to a concentration effect due to exclusion or a strong positive interaction with the air–water interface. We noticed that one of the molecules we imaged previously [12], namely human erythrocyte catalase, behaved anomalously. As the aqueous film became thinner during blotting, at a certain point the catalase molecules became concentrated by several hundred-fold compared with their expected concentration based on Avogadro’s number and the ice thickness.

Catalase is a relatively stable enzyme that is responsible for generation of oxygen from hydrogen peroxide. The name of the enzyme was coined in 1900 by Loew [14]. Subsequently, Warburg observed that cyanide inhibits this reaction and identified that the enzyme contains an iron atom. It was one of the first proteins to be crystallized [15] but the structural solution took more than four decades, with the structure of bovine liver catalase determined by X-ray crystallography in 1981 [16,17]. Three-dimensional crystals of catalase were also investigated by electron microscopy [18] and structure determination by electron diffraction became possible recently [19,20]. Catalase crystals have also been used in electron microscopy as a standard specimen for magnification calibration.

In this paper, we follow up with an in-depth study of the unusual behaviour of human erythrocyte catalase in the hope that what we learn from catalase might be more generally useful. Concentration effects upon blotting generally occur when the sample has a strong interaction with the air–water interface. During these investigations, we also obtained a cryoEM structure for human erythrocyte catalase whose cryoEM map and coordinates have been deposited in the Electron Microcopy Data Bank and Protein Data Bank, respectively.

## Methods and materials

Human erythrocyte catalase was obtained from Sigma (C3556) at a concentration of 2 mg/ml. It was either concentrated and the buffer changed to phosphate-buffered saline (PBS), pH 7.4 using an Amicon Ultracel 100k centrifugal filter spin column or diluted into PBS. NADPH (β-nicotinamide adenine dinucleotide 2′- phosphate reduced tetrasodium salt hydrate, Sigma-Aldrich, N7505) was added at 0.2 mM prior to concentrating. Detergents were added, normally slightly above the critical micelle concentration (CMC), after adjustment of catalase to the desired protein concentration. Dodecyl-β-D-maltoside (DDM) was obtained from Glycon (D97002). 1H, 1H, 2H, 2H-perfluorooctyl-phosphocholine (FC-8) and 1H, 1H, 2H, 2H-perfluorooctyl-β-D-maltopyranoside (FOM) were obtained from Anatrace (F300F, O310F). 3-(3-cholamidopropyl-dimethylammonio)-2-hydroxy-1-propanesulfonate (CHAPSO) was obtained from Sigma (C3649). 1,2-dihexanoyl-sn-glycero-3-phosphocholine and 1,2-diheptanoyl-sn-glycero-3-phosphocholine were obtained from Avanti Polar lipids, Inc.

Plunge-freezing was carried out in a cold room at 4°C using a controlled environment plunge-freezer [21]. Holey carbon grids (Quantifoil R 1.2/1.3 300 mesh Cu), after blotting and freezing, were loaded into a Thermo Fisher Krios and images recorded at nominal magnifications of either 59 000 × or 75 000 ×  on either a Ceta detector in integrating mode or a Falcon 3 detector in counting mode. These combinations produce pixel sizes of 1.4 and 1.07 Å, respectively. The dataset used for the structure determination consisted of 356 images/movies with 1.07 Å pixel size and the movies were stored as 75 frames with exposure of ∼0.5 el/Å^2^/frame. Beam-induced motion was corrected using MotionCor2 [22]. Defocus and astigmatism were estimated using CTFFIND4 [23]. All other steps of image processing were carried out with RELION [24]. Particle coordinates from the summed micrographs were picked using the Laplacian of Gaussian operator in RELION. A total of 150 000 particles were extracted with an initial box size of 150 pixels extended to 400 pixels later, followed by rejection of about 20% of particles using 2D and 3D classification. A three-dimensional density map was calculated from 119 000 particles, constrained to D2 symmetry. The map was of good quality with continuous density throughout. Orientation distributions, such as shown in Fig. 3, were analysed using the program cryoEF, which calculates a criterion for the efficiency of the orientation distribution, called Eod [6]. Model building using *Coot* [25] started from the 1F4J PDB coordinates [26], with addition of the N-terminus and NADPH ligand from 4BLC (bovine liver catalase [27]). All water molecules were introduced without reference to previous structures, using *Coot*’s ‘Find Waters’ and difference maps calculated by Servalcat [28]. 1DGF was not used to avoid any bias, except for a final comparison and to identify any regions that required further study [29]. Rounds of model building in *Coot* alternating with coordinate refinement in REFMAC [30] and all-atom contact analysis in Molprobity [31] led to the structure shown in Fig. 4 and Table 2. Figures were made using PyMOL (The PyMOL Molecular Graphics System, Version 2.0 Schrödinger, LLC) or Chimera [32].

**Fig. 1.**
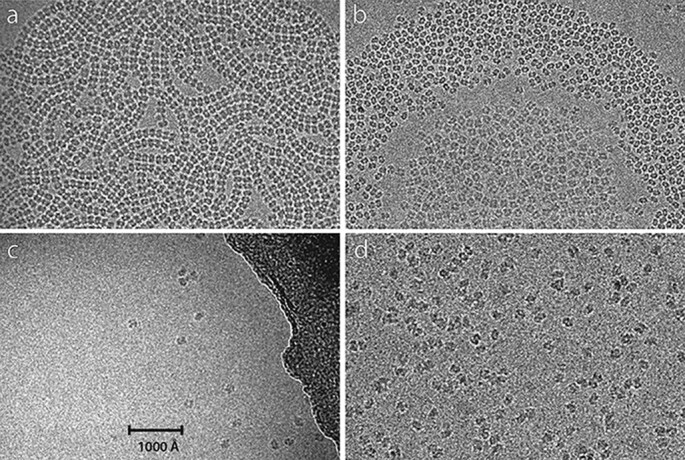
Behaviour of human erythrocyte catalase at the air–water interface. (a) Ribbons of catalase molecules are produced from 0.05 mg/ml catalase in detergent-free buffer when blotted to form a very thin film. The catalase molecules adopt a single ‘edge’ orientation with respect to the air:water interface and interact with one another to form the ribbons. (b) 0.1 mg/ml catalase in 0.2 mM DDM after blotting to form a thin film. In this case, the catalase molecules are still concentrated at the air-detergent-water interface but have a weaker interaction with each other. The molecules in the central region form a raft with their short axis oriented perpendicular to the film; a gap with very few molecules separates this central raft from another crowded region with molecules in an edge orientation. In the gap, a few molecules showing a dumbbell view are also seen. Outside these crowded central regions, very few molecules are found, which is consistent with their low bulk protein concentration. (c) 1 mg/ml catalase in 4 mM Fos-choline-8 shows few molecules in many different orientations but no rafts or ribbons. (d) 40 mg/ml catalase in 8 mM CHAPSO, with good distribution. The last two panels show a distribution of catalase molecules at approximately the expected level in the absence of any surface interaction.

**Fig. 2.**
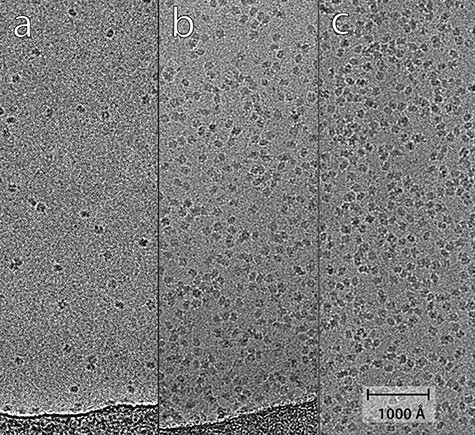
Panel showing how the observed number of catalase particles increases with protein concentration: (a) 1 mg/ml, (b) 10 mg/ml, (c) 25 mg/ml, all with 8 mM CHAPSO. A numerical comparison between the number of observed and expected particles per square micron is presented in Results, and shows good agreement.

**Table 1.** Calculated number of particles of molecular weight 230 000 expected in 4000 × 4000 Å square image area for a specimen thickness of 300 Å, provided depletion or surface adsorption is prevented

Catalase protein concentration (mg/ml)	Number of particles expected in images of a 4000 × 4000 Å area, 300 Å thick
0.1	1
0.2	3
0.5	8
1	15
2	30
5	60
10	125
20	250
40	500

**Table 2.** Molprobity analysis. PDB code: 7P8W

All-atom contacts	Clashscore^a^	1.58	100th percentile^b^ (*N* = 456, 2.20 Å ± 0.25 Å)
Protein Geometry	Rotamers favoured	1652	(95.38%)
	Rotamers poor	12	(0.69%)
	Ramachandran favoured	1920	(96.00%)
	Ramachandran outliers	4	(0.20%)
	Ramachandran distribution Z-score	1.36 ± 0.16	
	MolProbity score^c^	1.18	100th percentile^b^ (*N* = 10 167, 2.20 Å ± 0.25 Å)
	Cβ deviations >0.25 Å	4	(0.21%)
	Bad bonds	8/17 084	(0.21%)
	Bad angles	47/23 312	(0.20%)
Peptide Omegas	Cis Prolines	4/148	(2.70%)
Additional validations	Chiral volume outliers	0/2352	
	Waters with clashes	8/1112	

a Clashscore is the number of serious steric overlaps (>0.5 Å) per 1000 atoms.

b 100th percentile is the best among structures of comparable resolution.

c MolProbity score combines the clashscore, rotamer and Ramachandran evaluations into a single score, normalized to be on the same scale as X-ray resolution.

**Fig. 3.**
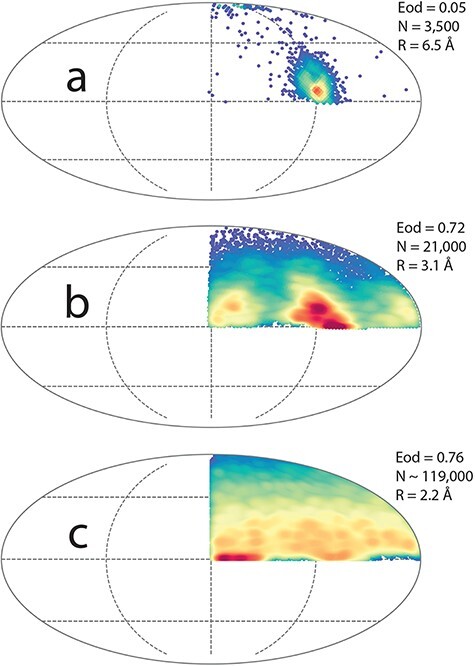
Orientation distribution for catalase grids prepared under different conditions. (a) Distribution found without added detergent showing one predominant orientation, (b) distribution in presence of 8 mM CHAPSO with very thin ice film and without added NADPH, (c) nearly optimal distribution in presence of 8 mM CHAPSO and added NADPH.

**Fig. 4.**
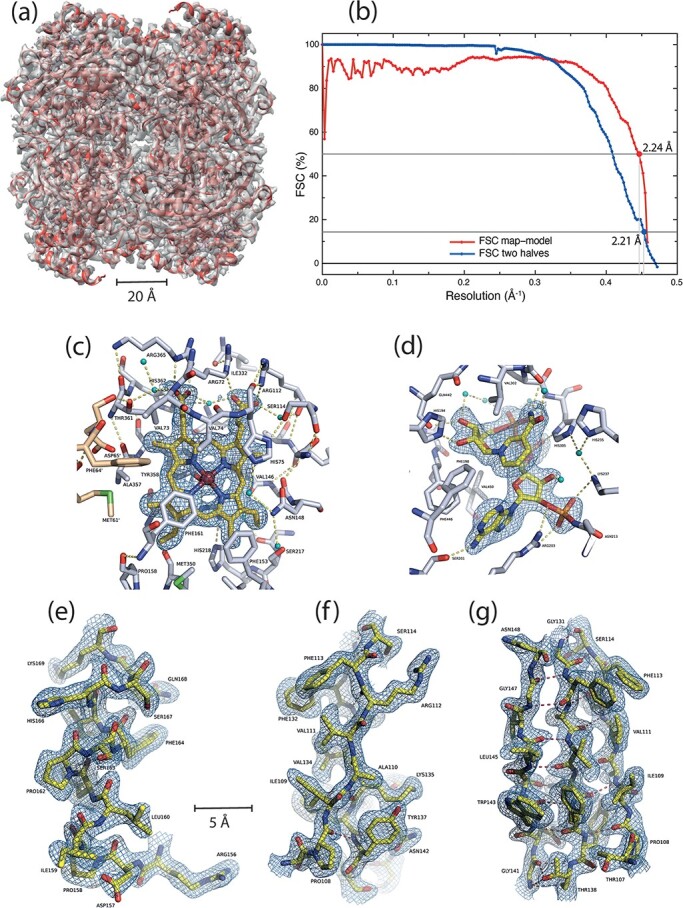
CryoEM structure of catalase obtained after minimizing the air–water interaction problem using detergent. (a) Overlay of the final model (shown in cartoon representation in red) and the final sharpened EM map in transparent gray. (b) FSC curves between two halves of data and between map and refined model. Parts of the map and corresponding model are shown: (c) near the heme, (d) at the NADPH ligand-binding site, (e) around α-helix 156–169, (f) side-view of a 3-stranded β-sheet with visible side-chains and (g) front view of the same 3-stranded β-sheet. The small blue-green spheres are water molecules.

## Results

Figure 1 shows a sequence of four panels, with (a) ribbons, (b) rafts and finally free non-interacting catalase molecules in panels (c) and (d), obtained after addition of either FC-8 or CHAPSO, with detergent concentrations just above their CMC. These images were obtained in the same order as presented in the following sequence.

Initially grids were prepared using catalase at 2 mg/ml as obtained from Sigma. Images from these showed overcrowding with far too many particles, often overlapped. Step-by-step dilution in PBS, to concentrations as low as 0.05 mg/ml allowed images like that in Fig. 1a to be obtained, showing extended close-packed ribbons of catalase in a single predominant orientation. This behaviour was unaffected by increasing or decreasing pH to 8.1 or 4.5, using Tris or acetate buffer, but adding mild detergents known to be well tolerated by many membrane proteins, such as DDM, changed the behaviour. The decreased surface tension in the presence of DDM produced consistently thinner ice, but in the thinnest regions also produced rafts of catalase molecules orientated with their short axis perpendicular to the surface of the film. Surrounding these rafts, an empty region separated the rafts from a ring of molecules in the same orientation as found in the ribbons, as shown in Fig. 1b. There was still a tendency to form ribbons but less prominently. Nearer the edge of the holes in the Quantifoil carbon film, where the ice is thicker, the number of catalase molecules observed was much lower, and in reasonable agreement with that expected for the low concentration used, such as 0.1 mg/ml in Fig. 1b. Because the number of molecules in the centre of the holes where the rafts and ribbons formed was up to one thousand times greater than expected from the bulk solution concentration, it was clear that the catalase molecules must interact strongly with both air–water interfaces, and that the interaction is modified by the presence of DDM. At this point, a wider range of detergents was explored, especially the perfluorinated FC-8 and FOM, as well as CHAPSO, which others had found useful either for stabilizing membrane proteins [33] or for preparing cryoEM grids by blotting and plunge-freezing [34] Fig. 1c and d show the expected numbers of particles based on protein concentration, allowing us to conclude that FC-8 or CHAPSO at concentrations just above CMC produced the desired, predictable particle distribution.

We then carried out a more quantitative analysis to compare the observed behaviour with that expected from a calculation based on Avogadro’s number and the estimated thickness of the ice layer, as shown in Table 1. Ice thickness was not measured directly, but was estimated from the images based on prior experience. Figure 2 shows three panels prepared with catalase protein concentrations of 1, 10 and 25 mg/ml, with added 0.2 mM NADPH and 8 mM CHAPSO. The number of particles visible in each of the three panels is approximately 25, 230 and 400, respectively. The calculations shown in Table 1 suggest there should be 18, 150 and 370 respectively assuming the ice is 300 Å thick, since the area shown in each panel is 7300 × 2600 Å^2^ compared with the 4000 × 4000 Å^2^ area for the calculations shown in Table 1. The observed and calculated numbers of particles agree fairly well (ratio of observed to calculated is between 1.1 and 1.5) noting that the ice might be slightly thicker than 300 Å near the edges of the holes in the carbon film. In the main dataset, the average number of particles extracted per micrograph was 420 and the average number used was 330. For a micrograph area of 4380 × 4380 Å^2^, the expected number from Table 1 would be 600, but given that overlapped particles and particles that were too close, as well as carbon film regions at the edge of holes, were excluded this shows satisfactory agreement between the observed and expected numbers of particles.

Many datasets were collected. Earlier (unpublished) efforts using grids with smaller holes to produce thicker ice to try to avoid the surface effects described above gave images with more inelastic scatter and lower contrast. These grids showed some improvement on the orientation distribution, resulting in 3D structures but with resolutions never better than ∼4 Å. As part of an investigation of the viability of 100 keV cryoEM, Naydenova *et al*. [35] obtained a 6.5 Å resolution catalase map, with molecules almost exclusively in one orientation, as shown in Fig. 3a. Figure 3 shows plots of the orientation distribution (program cryoEF [6]) of three different datasets collected from grids prepared under different conditions. In Fig. 3a using data from a grid prepared in the absence of detergent one predominant orientation occurs. That orientation corresponds to the edge view seen in the ribbons in Fig. 1a. With added 8 mM CHAPSO, the distributions shown in Fig. 3b without added NADPH and 3(c) with added NADPH, suffer from much less preferential orientation. The influence of NADPH on the distribution is quite small, but ensures that there is full ligand occupancy. The particles in Fig. 3c which had slightly thicker ice have the most isotropic distribution.

We also investigated whether the orientation distribution was affected by ionic strength, by reducing the NaCl concentration to 15 mM or increasing it to 350 mM. Small datasets of about 200 images/movies were analysed using RELION. There was no strong effect of ionic strength (Supplementary Fig. S1) either using CHAPSO, or dihexanoyl phosphocholine (DHPC-hexa) or diheptanoyl phosphocholine (DHPC-hepta) detergent additives, although there is a slightly more isotropic orientation distribution at lower ionic strength (Supplementary Fig. S1).

**Fig. 5.**
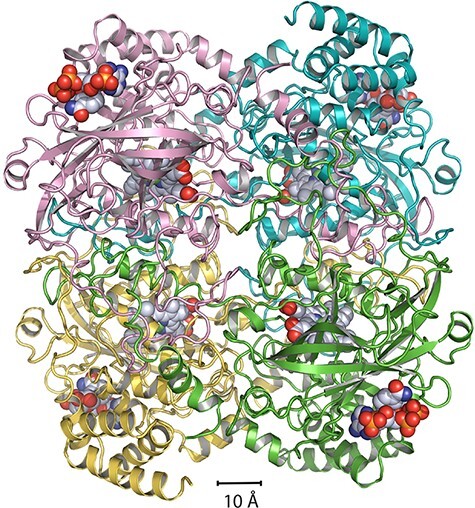
Schematic overview of the catalase tetramer to show the locations of the heme and NADPH (space-filling representation) in relation to the four subunits in the tetramer (helices and sheets as ribbons). The magenta, yellow, green and cyan subunits show the dimer of dimers structure with extensive strand exchange throughout.

The dataset used to collect images (and movies) for the map shown in Fig. 4 was obtained from a single grid made with a catalase concentration of 40 mg/ml in the presence of 8 mM CHAPSO, and 0.2 mM NADPH. After excluding a small proportion of particles using RELION 3D classification, the map and model shown in Fig. 4 was obtained from a single dataset of 119 000 particles, representing 80% of those picked initially using RELION’s Laplacian of Gaussian operator. The resolution was 2.21 Å at FSC = 0.143 between two half-sets and 2.24 Å at FSC = 0.5 between map and refined atomic model (Fig. 4b), which corresponds to 97% of the limiting Nyquist spacing for the 1.07 Å pixel size. An earlier dataset of 6900 particles using similar conditions reached 2.9 Å resolution. Example densities are shown in Fig. 4c–g. A schematic overview of the catalase tetramer to show the locations of the heme and NADPH in relation to the four subunits is shown in Fig. 5. The resolvability of atoms in the current map estimated with MapQ [36] is shown in supplementary figure S2 to be very good.

## Discussion

### Use of detergent to provide a barrier to separate protein from air

We have used detergents in this work as surfactants rather than to form micelles. We have used concentrations at or slightly above their CMC, yet the CMC is a measure of the detergent monomer concentration that represents the threshold for micelle formation. The detergent concentration for formation of a surface monolayer may differ from that needed for micelle formation, since the environment inside a detergent micelle is different from that of air at the surface. For CHAPSO, we used the pendant drop method [37] to measure the surface tension by comparing the weight of drops from the tip of a clean glass Pasteur pipette as a function of detergent concentration. The surface tension of PBS, assumed to be 72 mN/m, was reduced to a plateau of 42 mN/m (58%) by addition of CHAPSO at all concentrations above 2.5 mM. The concentration that produced half the maximum reduction was 0.5 mM. We were therefore assured that a robust surfactant monolayer was being formed, and that the concentration we used was adequate to produce the maximum effect, so that the exact conditions were not critical.

We ended up by using relatively high protein concentration after preventing interaction with the air–water interface using CHAPSO, to produce a good distribution of particle number and orientation. This approach is useful until a better method is developed. For example, one strategy might be to freeze a thicker layer of sample. This would have the advantage of reducing the surface-to-volume ratio during initial freezing, so that more molecules would have an environment closer to that of bulk solution. If the two surfaces were then removed by focussed ion beam milling (FIB-milling), that would keep the specimen thin enough for high resolution imaging. This approach has already been tested on FIB-milled lysozyme crystals, with retention of high-quality electron diffraction and subsequent structure determination by molecular replacement [38,39].

It would also be useful to explore a wider range of detergents or lipids with better properties such as stronger hydrophobic side-to-side interactions and more universally tolerated hydrophilic regions. Surface modification treatments in other fields are often described with the goal of passivating the surface. This is the term used in fluorescence or antibody staining methods, in which serum albumin is often used to pre-treat a surface to prevent subsequent non-specific binding or adsorption. In other instances, polyethylene glycols of various lengths are used, such as thio-PEG for passivating a gold surface [40–42], or linear and branched-chain amine-reactive PEGs to carry out dense polymer brush grafting for optical and fluorescence microscopy on derivatized glass surfaces [43].

No systematic exploration of the detergent properties needed for preparation of thin films for plunge-freeze cryoEM has been made, although there is some excellent recent work that has investigated several detergents and several different macromolecules [44,45]. There are useful reviews of protein denaturation at the air–water interface [46], and studies of surface tension reduction [47,48]. CHAPSO was found to be uniquely useful for preventing surface interaction and preferred orientation for several RNA polymerases [34], although CHAPSO molecules were also observed to bind to several sites in surface crevices on the protein. No adventitious CHAPSO binding was observed in our catalase map.

There are several factors that will be important for surface-active molecules for optimal use in cryoEM work. For example, the CMC, the micelle, vesicle or filament size, depending on the nature of the aggregates that are formed at higher concentrations, and the internal rigidity of the detergent monolayer are all likely to be important. It may be desirable to use a surfactant with higher CMC to ensure a rapid equilibration to the fresh surfaces that are probably formed during the blotting step with filter paper.

We have investigated only a small number detergents, including DDM, fluorinated-FC-8, fluorinated-C8-maltoside, DHPC (both dihexanoyl- and diheptanoyl-phosphocholine) and CHAPSO. These were chosen with the expectation that very hydrophilic headgroups, such as those with a zwitterionic structure could be most useful, and that short or medium length hydrophobic tails would keep a relatively high CMC so that equilibration would be fast. For catalase, the effect varying the pH between 4.5 and 8.1, or the ionic strength from 15 to 350 mM had relatively small effects. The analysis shown in Supplementary Fig. S1 shows that lower ionic strength for catalase results in a slightly more isotropic distribution of orientations. Glaeser and Han have published [13] a valuable opinion piece concerning the adsorption of proteins at the air–water interface, and the possible influence of surfactants in minimizing adsorption and preferential orientation. The observations we make here are in keeping with those views, and provide practical confirmation of the importance of obtaining a relatively isotropic orientation distribution, which was already clear from theory. The success of the limited range of detergents and conditions we have used suggests that more extensive investigations would be useful. In addition, very little is known about the nature and kinetics of how proteins might penetrate a surface amphipathic monolayer although this has been discussed [47]. Investigating this further could be very valuable in gaining a deeper understanding of plunge-freezing in cryoEM in the presence of detergent additives.

Some people might say that one could equally well plan to take advantage of any adsorption to the air–water interface, since this would allow the use of lower concentrations of protein. However, this can only be helpful provided the adsorption is isotropic and not accompanied by a preference for a limited number of orientations. That was certainly not the case for human erythrocyte catalase, and would seem to be at best a risky philosophy. The concentrating effect of the air–water interface may nevertheless have been beneficial in some cases.

### Structure of frozen hydrated catalase

The map we have obtained shows continuous density and excellent resolution from residue Ser-4 to Pro-505. A few residues at the C-terminus have slightly higher refined B-factors than the rest of the structure, consistent with being slightly less well-ordered. All atoms in the heme group and the NADPH ligand, including the three phosphate groups, are clearly resolved with no ambiguities. In comparison to the 502 residues in the current single-particle cryoEM model, the earlier X-ray crystal structure analyses contain 497 residues (1DGF), 498 residues (1DGB), 479–481 residues (1F4J) and 498 residues (3NWL), due to N-terminal or C-terminal disorder.

A comparison of the models of catalase from crystals and frozen hydrated single particles show that they are largely identical with only minor differences in some loop regions. The RMSD of aligned Cα atoms after secondary structure matching was 0.22–0.26 Å against different subunits of 1DGF, 0.28–0.31 Å against those in 1DGB, and 0.35–0.37 Å against those in 3NWL. So the cryoEM model at 2.2 Å resolution is more similar to the crystal structure at 1.5 Å resolution (1DGF) than the two others at the same or lower resolution. Since the 4BLC structure was determined at 290 K, that comparison was not made. An interesting aspect of catalase is it has a high affinity for the substrate NADPH, which is responsible for providing the reductive power to convert hydrogen peroxide into water. In the cryoEM maps, all four subunits have clear density for NADPH (Fig. 4d). Unlike other enzymes, where NADPH is bound in an extended conformation, the adenine and nicotinamide rings are brought closer and are at right angles to each other in catalase. In some of the X-ray structures, only two bound NADPH molecules are observed (1DGB, 1DGF, 1DGG, 1DGH). The exact reason why the NADPH molecules are not observed in all the subunits of the X-ray structures is not clear but could be due to crystallization conditions.

We have modelled 278 water molecules in each subunit of catalase based on the EM map. These are included in the PDB-deposited model but a higher resolution map would help a more detailed discussion, so we do not discuss them further.

## Concluding remarks

The behaviour and interactions of proteins or other macromolecules at the air–water interface is a critical factor in cryoEM. The addition of an amphipathic detergent, which forms a monolayer at the air–water interface at concentrations near or just above the detergent CMC, can provide a barrier in which the protein experiences the surface via the hydrophilic end of the detergent, rather than directly to air. From the limited number of detergents that we examined, it is clear that CHAPSO is better than others in this respect: it has a relatively high CMC and a fairly rigid hydrophobic core, and these two factors may explain why it is particularly effective. It would be valuable to carry out more systematic investigation of a wider range of detergents and to develop some underpinning theory. While the rate-limiting step in X-ray crystallography is obtaining good crystals, the rate-limiting step in cryoEM is increasingly going from purified protein to a single cryoEM grid with good particle and orientation distribution. Structure determination by single-particle cryoEM can then be very efficient. The use of higher protein concentration, such as the 25–40 mg/ml we used for catalase, gives the additional advantage of many particles per image, as well as having a relatively isotropic orientation distribution. The use of higher protein concentrations in the presence of detergent is a good approach while other methods are being developed, such as the use of affinity grids or derivatized graphene oxide [49–51].

## Supplementary Material

dfab037_Supp
