# Supplementary figures and images for: Interaction of human erythrocyte catalase with air–water interface in cryoEM
Source: Microscopy (Oxf). 2022 Feb 18;71(Suppl 1):i51–9. doi: 10.1093/jmicro/dfab037 (PMC8855524; doi:10.1093/jmicro/dfab037)

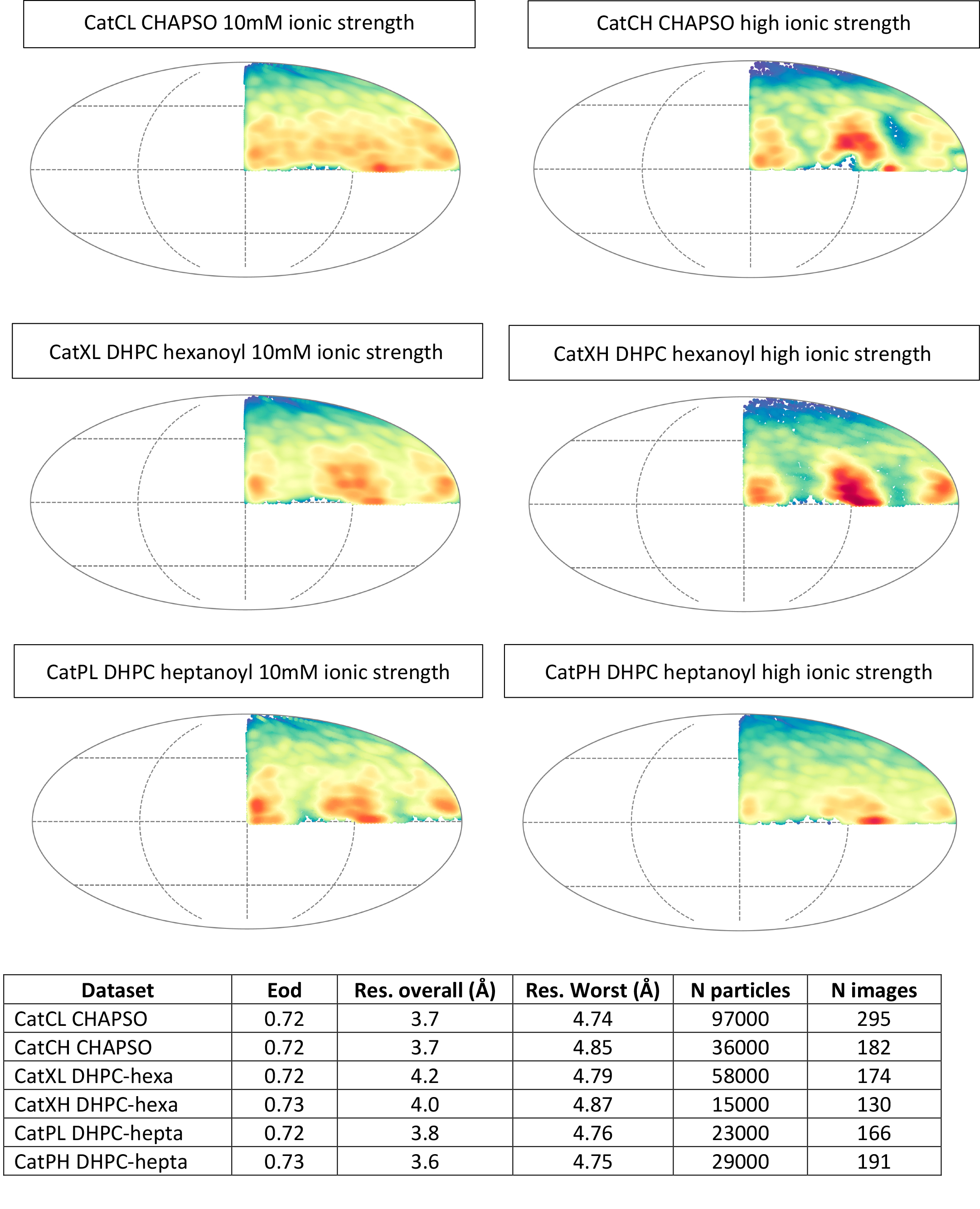

Supplement: dfab037_Supp [file dfab037_supp.zip › Fig_S1_catalase_orientation_6_plots_3_detergents_for_paper_v2.jpeg]

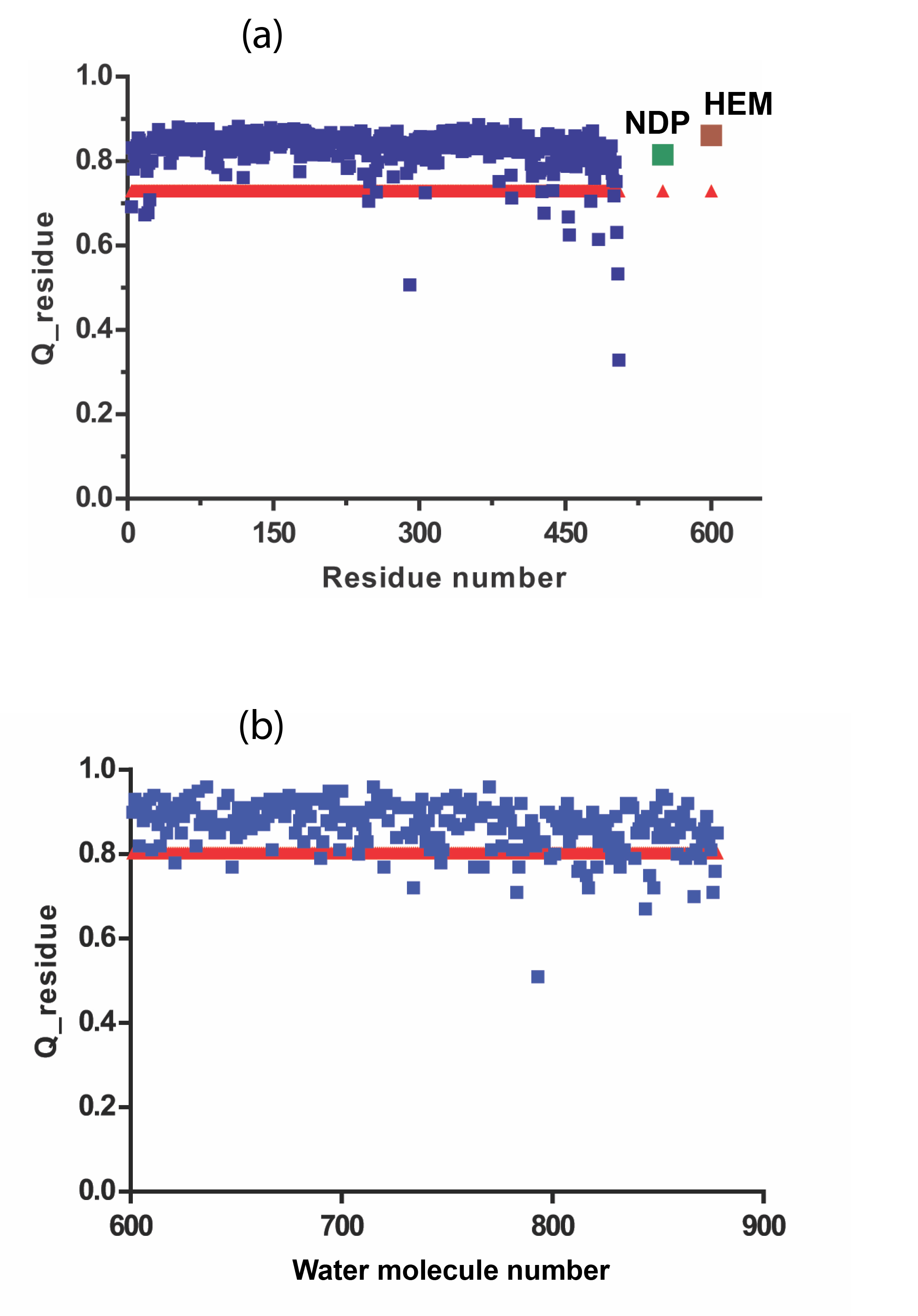

Supplement: dfab037_Supp [file dfab037_supp.zip › Final_Fig_S2_Q_residue_plots.jpg]
